# Kikuchi-Fujimoto disease, case report

**DOI:** 10.1093/omcr/omae211

**Published:** 2025-03-28

**Authors:** Juan M Castañeda Martinez, Jose J I Y Zamora Diaz, Alison A Rodriguez Avila, Sandra B Somarriba Dominguez, Luis A Nava Fuentes

**Affiliations:** Cardiology, CMN 20 de noviembre ISSSTE/ Universidad Nacional Autonoma de México, 03100, México; Cardiology, CMN 20 de noviembre ISSSTE/ Universidad Nacional Autonoma de México, 03100, México; General medicine, Universidad Autonoma de Guerrero, Lázaro Cárdenas 88, Guerrero, 39086, México; Cardiology, CMN 20 de noviembre ISSSTE/ Universidad Nacional Autonoma de México, 03100, México; General medicine, Universidad Autonoma de Nayarit, Boulevard 325, Nayarit, 63180, México

**Keywords:** Kikuchi-Fujimoto, histiocytic necrotizing lymphadenitis, case report

## Abstract

Kikuchi-Fujimoto disease (KFD), or histiocytic necrotizing lymphadenitis, is a rare, benign, and self-limiting condition characterized by subacute necrotizing cervical lymphadenopathy. Diagnosed through excisional lymph node biopsy, KFD predominantly affects young adults, especially females, with a higher incidence in Asian populations. The case discussed involves a 17-year-old male from Nayarit, Mexico, presenting with recurrent cervical lymphadenopathy unresponsive to antibiotics. Physical examination and imaging suggested lymphoproliferative disease, while biopsy confirmed histiocytic necrotizing lymphadenitis. Immunohistochemistry revealed CD68 positivity. KFD’s aetiology is unclear, with infectious and autoimmune theories proposed. The disease commonly resolves within 4 to 6 months with symptomatic management, including antipyretics and analgesics. This case is notable due to the patient’s young age, large lesion size, and male gender, diverging from the typical presentation, underscoring the importance of considering KFD as a differential diagnosis to avoid unnecessary invasive procedures.

## Introduction

Khi-Fujimoto disease (KFD), also known as histiocytic necrotizing lymphadenitis, is a relatively rare condition characterized by subacute necrotizing cervical lymphadenopathy, being a benign and self-limiting disease [1]. It was first described in 1972 by the Japanese scientist Seishi Kikuchi, who identified it as a rare benign disorder and independently reported by Y. Fujimoto in the same year. The disease commonly causes mild fever, tender cervical lymphadenopathy, night sweats, and headache [2]. The diagnosis is confirmed by performing an excisional lymph node biopsy. This biopsy typically shows a low number of neutrophils and eosinophils. Immunohistochemistry reveals histiocytes positive for myeloperoxidase and CD68, CD8-positive T cells and infrequent B cells [3]. KFD primarily affects young adults, with an average age of 20 to 30 years, and has a higher incidence in females [4, 5].

Case report: A 17-year-old male from Nayarit, Mexico, with no history of chronic degenerative diseases. He has a positive history of smoking for five years and alcohol use. The condition began in 2019 with swelling on the left lateral side of the neck, which resolved with unspecified antibiotic therapy without associated symptoms. In 2020, he experienced another increase in the size of the left lateral neck area, which did not respond to antibiotics, and denied associated symptoms. The patient was referred to the internal medicine department for additional evaluation.

On physical examination, vital signs were as follows: blood pressure 120/80 mmHg, heart rate 80 bpm, respiratory rate 18 breaths per minute, temperature 36.5°C, weight 66 kg, height 1.72 m. The patient was neurologically intact, with appropriate skin colouration. The chest was symmetrical with normal breath sounds, rhythmic heart sounds without abnormalities, and an abdomen with normal peristalsis. Extremities were intact and symmetrical. In the neck, there was no jugular venous distention; a soft, non-mobile mass with regular borders and slight tenderness was observed on the left lateral side.

Admission laboratory results included haemoglobin 15.1 g/dl, leukocytes 6.5 × 10^3/μl, neutrophils 66.8%, platelets 336 000/μl, creatinine 0.7 mg/dl, urea 18.9 mg/dl, and glucose 93 mg/dl. A neck ultrasound reported a conglomerate of left cervical lymph nodes, specifically in levels IIA and III, with a high suspicion of either an infectious or lymphoproliferative process.

**Figure 1:**
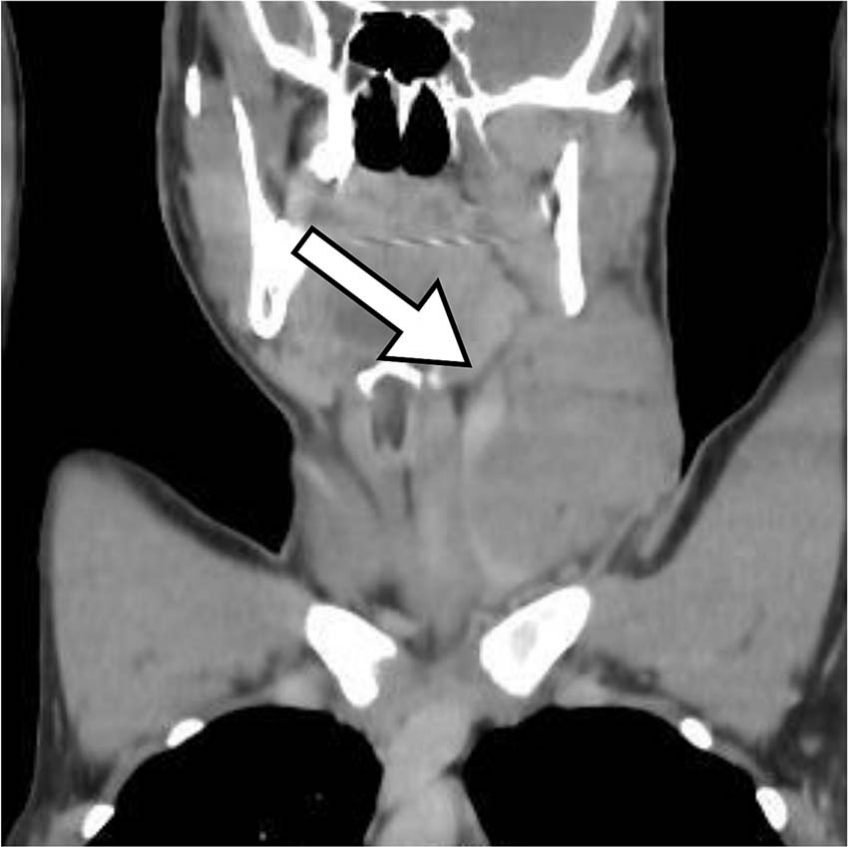
Coronal section of CT -neck showing supraclavicular lymph node (white arrow).

A chest and abdominal CT scan revealed a left supraclavicular lymph node conglomerate, with enlargement of mediastinal lymph nodes (Fig. 1).

**Figure 2:**
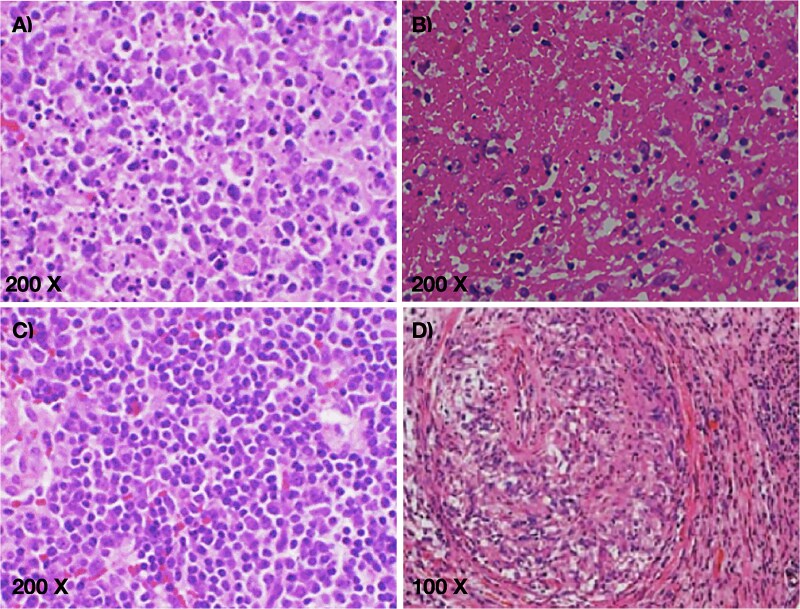
Hematoxylin and eosin-stain (A-D). Microscopic images of cervical lymph node biopsy: A) acute inflammatory process with infiltration of mature lymphocytes, neutrophils, and apoptotic bodies. B) Necrotic material with a high level of fibrin and cellular debris. C) Areas of proliferation of plasma cells and epithelioid histiocytes. D) Lymph node vessels with a muscular wall infiltrated by lymphocytes, resulting in vascular lumen obliteration and wall thickening, highly suggestive of autoimmunity.

A biopsy of the left cervical lymph node showed a non-neoplastic lesion with the presence of histiocytic necrotizing lymphadenitis, negative for malignancy (Fig. 2).

**Figure 3:**
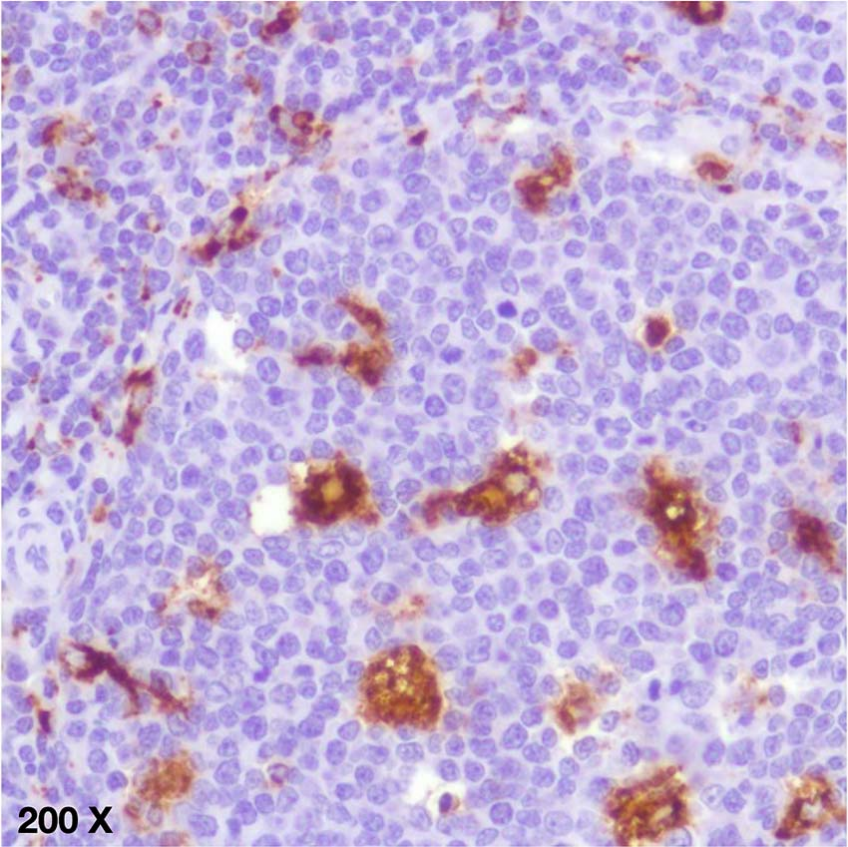
Immunohistochemical study with bio SB antibodies, 1:500 dilution, antigen retrieval by heat. Staining was performed using the avidin-biotin technique with diaminobenzidine and hematoxylin. CD68 antibody staining shows intense membranous positivity in clone L20.

An immunohistochemical study using CD68, CD123, and myeloperoxidase antibodies showed positivity for CD68 clone L20—immunohistochemical diagnosis of Necrotizing Histiocytic Lymphadenitis (Fig. 3).

## Discussion

The aetiology of Kikuchi-Fujimoto disease (KFD) remains unclear, with two accepted theories explaining its origin. The infectious theory suggests that various viruses and bacteria are responsible for triggering an exaggerated immune response in the host, with the main agents being Epstein–Barr virus, herpes simplex virus, varicella-zoster virus, herpesviruses 6, 7, and 8, parvovirus, HIV, Brucella, and Toxoplasma. On the other hand, an autoimmune response mediated by T cells is considered part of the disease’s pathophysiology. Compared to the general population, patients diagnosed with KFD more frequently possess the HLA-DPA1 and HLA-DPB1 genotypes [4, 6].

The population most affected by this condition are Asian patients, which aligns with the number of cases reported globally. The previously discussed case involves a Latin American patient without family connections to the Asian continent.

The patient’s age aligns with the literature, as it is within the 20–30 years range; however, a higher incidence has been observed in female patients, as reported by Huang C.J. and colleagues, making presentation in males even less common.

The typical location for this lymphadenopathy is unilateral cervical, which matches the case reported here. Computed tomography observed a mass size of 9 × 7 cm. The lesion’s dimensions exceed the average reported size of 1–4 cm in documented cases, and it is consistent with fever as the primary associated symptom [7, 8].

The definitive diagnosis of the lesion is made through tissue microscopy, with three proposed histological patterns: proliferative, necrotizing, and xanthomatous. The necrotizing pattern is characterized by cellular debris, various stages of apoptosis, and numerous perinuclear vacuoles, the phase observed in this patient. Immunohistochemical assays are positive for CD68, CD163, and CD4; however, this diagnostic method is reserved for previously inconclusive results.

Since it is considered a self-limiting disease, treatment is generally limited to symptomatic management. The main drugs used are antipyretics and analgesics. The natural course of the disease indicates remission within 4 to 6 months after diagnosis, which coincides with the progression observed in our patient.

## Conclusion

We present the case of a 17-year-old Latino male who developed Kikuchi-Fujimoto disease (KFD) without any prior reported infectious events. The lesion’s size significantly exceeded the average diameter of typical cases without causing significant alterations in respiratory mechanics. This is one of the few reported cases in the Latino male population. Highlighting these cases allows the medical community to consider KFD as a differential diagnosis in similar situations, reinforcing the condition’s benign nature and avoiding risky surgical procedures with high technical complexity.
